# Environmental Health Responses to COVID 19 in Western Australia: Lessons for the Future

**DOI:** 10.3390/ijerph19159393

**Published:** 2022-07-31

**Authors:** Jacques Oosthuizen, Melissa Stoneham, Toni Hannelly, Edmore Masaka, Giverny Dodds, Victor Andrich

**Affiliations:** 1 School of Medical and Health Sciences, Edith Cowan University, Joondalup, WA 6027, Australia; e.masaka@ecu.edu.au; 2 Public Health Advocacy Institute of Western Australia, Curtin University, Bentley, WA 6102, Australia; m.stoneham@curtin.edu.au (M.S.); t.hannelly@curtin.edu.au (T.H.); 3 University of Queensland, Herston, QLD 4006, Australia; givernyd@gmail.com; 4 Environmental Health Australia (WA), Midland, WA 6936, Australia; vic.andrich@eh.org.au

**Keywords:** COVID-19, environmental health response, professional recognition, public health policy

## Abstract

The COVID-19 pandemic tested the health system of Western Australia (WA) and the relatively new overarching legislative framework that guided the state-wide public health response to the pandemic. This study aimed to evaluate the experiences and roles of environmental health officers (EHOs) in WA during the management of COVID-19 and to identify any policy changes that are needed to facilitate the rapid deployment of EHOs during a future public health crisis. An online survey with 78 respondents was administered and analysis was both qualitative and quantitative. It was found that participants believed there was inadequate resourcing, workforce shortages, increased workloads, and a lack of recognition and integration of the profession within the COVID-19 response. Notably, more than 65% of the respondents believed they could have been better utilised during the pandemic. This research has demonstrated that the COVID-19 pandemic in WA had clear gaps in its processes for managing responses and resilience to pandemics. Policy recommendations outlining a more efficient and integrated delivery of environmental health services throughout the state during emergencies are discussed.

## 1. Introduction

Western Australia (WA) has a relatively new body of legislation, the *Public Health Act 2016* (the Act) [1], which provides the legal framework for the delivery of public health in the state. One of the key professional groups charged with duties under this Act are environmental health officers (EHOs), who are a critical workforce tasked with minimising or mitigating environmental threats to human health. Their role is to identify and characterize health risks and propose an approach to resolving issues to prevent people from becoming ill or injured. The World Health Organization (WHO) [2] estimates that 15 of the extra 20 years of life that we now enjoy, compared with a century ago, can be attributed to environmental health interventions. The public health function of local governments (LGAs) in WA regarding public health is defined under Part 2, Divisions 2, 3 and 4 of the Act [1]. LGAs are empowered to appoint EHOs as authorised officers to perform functions such as environmental health risk assessments, licensing, regulation, and the inspection of a range of premises, in particular, those that are regarded as high risk. These include businesses that prepare and serve food, especially to vulnerable populations, and premises where there is a risk of infection such as body piercing, tattooing, and aquatic facilities. In addition, local government EHOs assess public building safety and related emergency evacuation requirements, investigate public health nuisances, and are frequently involved in managing food poisoning cases and food recalls (*Public Health Act 2016*, Part 2, Division 3–5)).

The first cases of novel severe acute respiratory syndrome coronavirus 2 (SARS-CoV-2), later renamed Coronavirus disease 19 or simply COVID-19, were reported in December 2019, from Wuhan, China. The disease spread rapidly worldwide, causing a global pandemic [3]. By the end of December 2021, 5.94 million deaths had been reported worldwide; however, modelling has shown that excess mortality attributable to COVID-19 was 18.2 million (95% uncertainty interval 17.1–19.6) [4]. Australia, as a fairly isolated continent, managed to contain the pandemic better than most other countries, and as of 28 June 2022 there had been 9,185,633 cases with 11,304 deaths (0.1%) [5].The EHO workforce is employed across a variety of sectors; however, the majority are employed with either state or local governments. During the pandemic, the primary COVID-19 management roles for the EHO workforce employed within the State Government sector, focused on managing COVID-19 risks for large events, leading the review into ventilation risks in quarantine hotels, and participating in the SARS-CoV-2 wastewater testing program.

EHOs employed at the local government level form a public health workforce, deployed throughout the state, who have local knowledge and an understanding of the communities they serve. EHOs in WA have accredited tertiary qualifications that include units of study in epidemiology, communicable disease prevention and control, and they are skilled at assessing health risks and implementing strategies to mitigate those risks within a legislative framework. Local government EHOs are well placed for rapid deployment throughout the state at a community level to respond during a public health disaster such as an epidemic. However, during the COVID-19 pandemic, the WA EHO workforce was underutilized, as many public health functions were delegated to the WA Police (WAPOL).

This research aimed to evaluate the experiences of EHOs in WA and assess their roles in the management of the epidemic during 2019 to 2021 and to identify any policy changes required to facilitate rapid deployment of EHOs during any future public health crisis.

## 2. Materials and Methods

This mixed-methods study employed both qualitative and quantitative analysis. Ethical approval was obtained from the Edith Cowan University Human Research Ethics Committee (2020-02037). EHOs throughout metropolitan and regional WA were sent emails from Environmental Health Australia (EHA) (WA) and the Metropolitan Environmental Health Managers Group (MEHMG) inviting participation in an anonymous online survey. Surveys were IP specific so could not be undertaken more than once per computer. The survey, hosted by Qualtrix, was open for three weeks during July 2021, with weekly reminders sent to encourage survey completion. Participation was voluntary and no incentives for participation were provided. The survey link was distributed to over 350 Environmental Health practitioners, and 78 valid responses were received (response rate ~22%).

The survey tool used was based on the National Environmental Health Association (NEHA) of the USA, ‘Environmental Health Workforce Needs assessment in Response to COVID-19′ survey and the 2004 South Australian Environmental Health Australia ‘Environmental Health Workforce Attraction and Retention Review’. Environmental health researchers in South Australia (SA) adapted the survey for administration to EHOs in SA and it was then further modified for use in the WA context. The tool included 28 questions related to demographics, length of service in the profession, workforce issues, and the impact of the COVID-19 pandemic. The tool contained both open-ended and closed questions.

Logistic regression and ordinal logistic regression methods were used to identify any relationships with covariates using a *p*-value of 0.05 to determine significance. The open-ended questions were coded, and themes derived. Given the research aims, it was considered important to expand on these themes and gather more data to create a narrative describing the changes evolving within the environmental health profession due to COVID-19. Follow-up interviews were conducted with eight EHOs from both local and state governments who indicated their willingness to be interviewed in the survey. This allowed the researchers to explore EHO’s perceptions and values regarding the legislative framework and changes in roles throughout COVID-19. The interview was guided by the validated tool used for similar research in SA and the United States and contained seven open-ended questions. The interviews were conducted via an online portal and recorded.

All open-ended survey responses and interview data were transcribed and imported into QSR NVivo (Version 11) software, (qsrinternational.com) and analysed using thematic analysis [6]. An initial list of 17 codes was developed and trialed on two transcripts. A meeting of the researchers to compare the application of coding and to clarify node definitions was held. A codebook was developed. This process was repeated on one further transcript and consensus on the coding application was achieved. Two researchers then independently coded all remaining transcripts, meeting to discuss any emerging patterns and confirm the application of codes. The 17 codes were then nested into six macro themes and subthemes by examining connections between themes and considering how frequently themes were expressed. Both researchers independently summarised all codes and synthesised and organised thematic findings.

## 3. Results

This study collected both quantitative and qualitative data that allowed for a mixed method approach to data analysis.

### 3.1. Quantitative Survey Data

As shown in Table 1, more than half of the respondents (59%) were aged 40 or over, and the younger cohort of EHOs (<40) were mostly female (60%).

Most participants (94.9%) held undergraduate or post graduate qualifications in environmental health. Seven EHOs (8.9%) advised that they had obtained qualifications overseas. Most respondents (51.3%) had been employed at their current workplace for more than five years and 85.9% were in permanent full-time positions.

More than three-quarters (78.2%) of the respondents worked in the Perth metropolitan area, 21.8% were employed in regional areas, three (3.8%) were employed by the State Government and one (1.3%) was employed in the private sector. Most participants (42.3%) were employed in smaller local governments with five or fewer EHO positions. This distribution reflects the general profile of local government in WA, with most officers being employed by small metropolitan LGAs.

#### COVID-19 Related Work Impacts

The survey explored EHO’s perceptions related to personal safety concerns, deployment to tasks outside of their normal duties, and the adequacy of training to deal with COVID-19. EHOs who worked part-time were 16 times less likely to agree that they had access to adequate EH COVID-19 response technical information or guidance than EHOs who worked full-time hours; however, this result was not statistically significant (*p* = 0.027).

**Figure 1:**
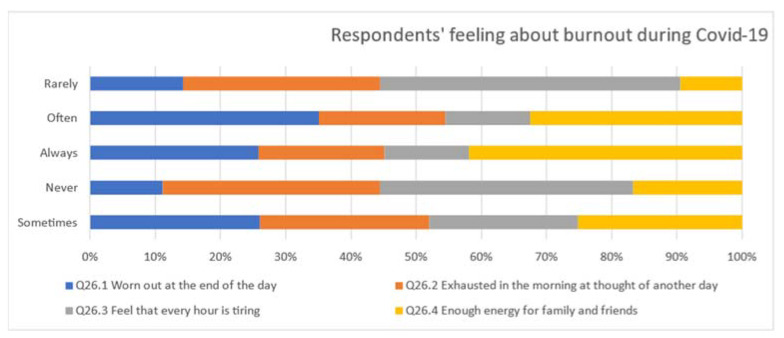
Feelings of burnout during COVID-19.

Participants were asked a series of questions comparing their perceptions of their workload before and during COVID-19, as well as their perceptions of professional burnout before and during COVID-19. Burnout has been described as the level of both psychological and physical fatigue perceived by an individual as this relates to their work [7,8]. In response to the questions “do you feel worn out at the end of the working day?” and “are you exhausted in the morning at the thought of another day at work?”, most EHOs (85.9% and 57.7% respectively), replied either “sometimes”, “often”, or “always”, and although not statistically significant (z = 0.329, *p* = 0.371), indicated that they were less worn out during COVID-19 than before (refer Figure 1).

As shown in Figure 1, EHOs stated that during COVID-19, they had less energy available to direct to family and friends compared to pre-COVID-19 (*p* < 0.024). This correlates to individuals being 2.4 times less likely to have enough energy for family and friends during leisure time than before COVID-19.

When respondents were asked if they felt they could have been better utilised in the response to COVID-19, 65.4% said yes, 33.3% said no, and 1.3% did not respond. Of the EHOs who felt they were underutilized, 59 (75.6%) provided further details in an open ended question. Table 2 provides a list of additional roles that EHOs could have assisted with, indicating that contract tracing, quarantine audits and risk communication were the most common responses.

More than one third (38.4%) of the respondents commented on the lack of integration or recognition of the environmental health profession within the COVID-19 response with an additional 5.1% stating that they felt there was a lack of authority afforded to them under the legislation.

### 3.2. Quanlitative Analysis

To extrapolate the survey data, interviews were conducted with eight survey respondents, and the interview data were used to provide context and depth. As detailed in the methods, respondents were asked to contextualise their answers or provide additional comments on the survey and during interviews. The resultant data were rich and illustrative. The process of repeated coding and re-coding resulted in six macro codes which served as a framework to analyse the comments. These macro themes and associated sub-themes are detailed in Table 3.

When asked if EHOs felt they could have been better utilized in the COVID-19 response, 65.4% agreed. These participants were invited to expand on their reasons for feeling that way. Comments around this theme saw EHOs perceiving their role as under-recognised and under-appreciated throughout the pandemic, with substantial powers being allocated to the WAPOL. Given that EHOs are tertiary educated in public health and emergency management, there was angst among participants, with numerous comments such as “we had very limited involvement due to restrictions on who is an authorised officer (role mainly taken up by WAPOL). This was frustrating, as EHOs are best placed to provide public health related delivery with respect to infectious disease advice and contact tracing.” Other participants expressed concern that “the government didn’t realize what we could actually offer” and another stating that “health ignored the profession” resulting in the profession being bypassed. Overall, comments such as this summed up the narrative in this theme; “I don’t think EHOs could have done everything, but we probably could have been a little bit more involved.”

The respondents were asked why they thought the environmental health profession was overlooked. Only eight (10%) felt this was not the case, while 24% felt strongly that they were undervalued, and the remaining 66% reported sometimes feeling undervalued. Many suggested that the professionals themselves or their professional association were to blame, with comments including “we are our own worst enemy. We sit on our hands. We don’t continue to learn. We don’t self-promote” Others felt that a more collective response was required, with the following comment illustrating this; “I guess it’s the ongoing battle with recognition and making sure we are marketing ourselves as environmental health professionals really well. I think it feels like we are a little bit forgotten.”

In contrast, a smaller number of respondents (10%) stated they were well utilized during COVID-19, with roles including the provision of information and dealing with enquiries from the public as well as business, the checking of compliance of businesses during lockdown, QR scanning and contact register compliance, and overseeing COVID-19 plans for events and providing guidance regarding sanitizing and cleaning as well as with regard to personal protective equipment (PPE). Other respondents reported that they were involved in developing health promotion resources showcasing preventive measures and the importance of vaccination [9]. One respondent advised “my role completely changed. I didn’t do any of my core environmental health officer duties. I was basically managing an emergency response group full time.” Another spoke of their solid educational foundation being fundamental in providing COVID-19-related advice within their organisation, stating “I just thought it was a bit like well no one else has this skill set that I have or the knowledge that I did, and I think because I had management experience in the past and emergency response experience in the past I think it was all just a natural fit to sit on the advisory committee.”

Given that most respondents felt they could have been better utilised during COVID-19, the interviews were used to elaborate on how this could have evolved. Although some respondents commented on the shift in roles, most felt they had the skills and potential to perform swabbing and contract tracing, which went unseen. Comments such as “I would have thought we would have been more involved in contact tracing, particularly locally. We probably would have been very well placed to help with that”, and “swabbing people or doing rapid antigen testing is probably not beyond the realms of our skills” illustrate this point.

The survey invited respondents to identify if they had access to adequate COVID-19 situational reports/updates and COVID-19 response technical information or guidance resources, and only three EHOs (4%) advised that they lacked technical information. However, when probed about this in the interviews, the respondents’ frustration was evident, with statements such as “our directions would come out two days after a public announcement”, and “we needed more advice on what EHOs could do to help their communities—like awareness.” A narrative around the clarity of the directions and advice was also clear, with comments including “we spent a lot of time trying to interpret the directions and read between the lines”, and “[a] direction would come out, but you would have to ask how it should be applied in this circumstance or community.”

A key research question was to identify the legal framework that governed the work of EHOs during the pandemic. Unfortunately, the survey did not specifically elicit this information, however as reported, over 20% of EHOs did not believe that they had been provided with the authority to act. When asked this question in the interviews, all eight respondents advised they did not use any regulatory tools during COVID-19. Some felt that although the *Public Health Act 2016* designated the chief health officer as the lead, they did not see this leadership or support. Others stated that “EHOs should have been authorised under the *Emergency Management Act 2005* to deal with the COVID-19 response at the community level” and others felt that EHOs had not been provided with “enforcement tools to see whether a venue was complying with COVID directions.”

The declaration of the COVID-19 emergency was made under both the *Emergency Management Act 2005* and the *Public Health Act 2016*, and this led to confusion about role delineation. Declaring the emergency under the *Emergency Management Act* meant that WAPOL had most of the powers. In addition, throughout the pandemic, most directions were made under the *Emergency Management Act* rather than the *Public Health Act*. This confusion resonated with the EHOs, with one respondent advising that within the *Public Health Act 2016* “it wasn’t clear if EHOs would have a full suite of new powers to cope with a future pandemic.”

Both the survey and interview questioned the respondents about human resources during the pandemic. It was identified that 61% felt their departments were understaffed. Many comments referred to the negative impact of poor resourcing on the EHO’s feelings of frustration and stress. Frequent themes were a shortage of qualified EHOs available to hire, a lack of EHOs on staff, a lack of time, and the lack of a regulatory framework. Many spoke of the increasing burden of the COVID-19 related tasks with one respondent stating, “we were checking all businesses for COVID registers with no more staff, so something had to give.” The shortage of experienced qualified Environmental Health Officers due to cuts to the tertiary sector, financial and other pressures experienced largely by local government was highlighted with comments such as “the actual numbers of EH officers to do the work is low. The number of people needed to address a pandemic is huge—just how are we going to get access to that?” Others reinforced a previous finding relating to the invisibility of EHOs, stating that “there are not enough EHOs, and they aren’t being appreciated enough in local government.”

## 4. Discussion

Workplace burnout has three key dimensions, including overwhelming exhaustion, feelings of cynicism and detachment from the job, and a sense of ineffectiveness and lack of accomplishment [10]. This research identified that, overall, the EHO participants identified that burnout was a workplace hazard, even before the COVID-19 pandemic. Although burnout can occur in any profession, it has been reported to be more common in professions such as human services, education and health care, where service relationships require an ongoing and intense level of personal and/or emotional contact [11]. EHOs experience these pressures with members of the business and community sectors as well as within their own organisations. This, combined with the finding that the EH workforce has been affected by various social, political, and economic factors such as an inability to recruit or retain staff, insufficient EHOs to complete regulatory work and funding cutbacks or policy restrictions, has resulted in work settings that are high in demand and low in resources, with a depleting number of EHOs to meet the demands of the job across WA.

The participants who were interviewed reinforced this hazard and reported that burnout rates were due to the stressful and emotionally demanding nature of the job, both before and during COVID-19. Research [9] suggests that the prevalence of burnout can have an impact on staff retention, and given the already low numbers of qualified EHOs available in the market, this is a concern that needs further investigation. Further research is also needed to consider the extent of burnout in the EH workforce and whether it is affecting community health outcomes or workforce issues including retention or retirement.

This research outlined that throughout the COVID-19 response, EHOs were undervalued and not utilised within collaborative teams to manage the pandemic. Notably, more than 65.4% of the respondents believed they could have been better utilized, citing duties such as quarantine audits, contract tracing and risk communication as being reasonable expectations. This is higher than that found in a similar study in South Australia, where approximately half of the EHO workforce believed that they could have been better utilised throughout COVID-19 [12]. This finding aligns with work conducted prior to the pandemic by Whiley et al., who undertook a media analysis and found that the term “environmental health officer received the least number of search results compared with other health professionals such as doctors, audiologists, occupational therapists and local government town planners [13].

Comments from participants identified that both the professionals, their professional association and, in some cases, their employers, needed to recognise more fully, the role and importance of the EH profession. As preventive health professionals, the benefits of EHOs doing their jobs successfully are likely to result in an issue never becoming a problem [14] or being recognised in the wider community. A good example of this in WA is trachoma, which almost entirely occurs in remote Aboriginal communities [13]. Australia is the only high-income country with endemic trachoma. The prevention of trachoma in these remote communities has been challenging. During the 1970s, the Australian Government treated nearly 40,000 Australians affected with trachoma [13]. In 2000, a review of 19 trachoma studies selected from the 39 conducted in different parts of the world showed that there is clear evidence to support the recommendation of facial cleanliness and environmental improvements to prevent trachoma [15]. In November 2006, the National Trachoma Surveillance and Reporting Unit (NTSRU) was established to combat trachoma among remote Australian Aboriginal communities, and instigated a national screening program of children five to nine years of age [16]. These data provide guidance on trachoma prevalence rates and locations. In 2019, the NTSRU stated that endemic levels of trachoma and poor facial cleanliness can only be addressed by expanding beyond screening and comprehensively implementing all aspects of the Surgery, Antibiotics, Facial hygiene and Environmental (SAFE) strategy by strengthening of health promotion strategies, environmental health improvements and health service activities [13]. This nineteen-year gap between the WHO advice that identified environmental health as a priority, and the NTSRU statement that comprehensive environmental health programs were needed, demonstrates the under-recognition of the environmental health profession in response to a public health condition.

The state government response to the COVID pandemic demonstrated a lack of understanding of the role of environmental health professionals. The centralised nature of decisions by non-environmental health qualified and experienced professionals missed a valuable opportunity to utilize the skill set of EHOs. This lack of understanding was reflected in the local government sector, where it was reported that some jurisdictions, during COVID-19 lockdowns, were going to stand down their environmental health workforce because they were not specifically called upon to participate in the response.

There is a substantial difference in how EHOs were utilised during COVID-19 in the United Kingdom compared to Western Australia. A survey of EHOs from English district councils, metropolitan district councils, unitary authorities and London boroughs between November 2020 and February 2021 was conducted, and received 211 responses. Of the EHOs redeployed in the pandemic, 98% were involved in enforcing business restrictions, 97% assisted in advising businesses on trading safely, 95% developed COVID policies and procedures, 78% managed local outbreaks, 69% were involved in emergency planning, and 59% were involved in contact tracing. Others were involved in community support programs such as food banks [17].

As noted by Maipas et al., in 2021, the ongoing pandemic may be associated with significant environmental health hazards that need continuous risk analysis and management through collaboration with all relevant stakeholders [18]. This study identified that such collaboration did not occur in WA, with most of the primary response and powers associated with managing the pandemic being offered to the police. Given that COVID-19 is a potentially fatal disease emerging from the interface between animals and humans [19], it would seem sensible to have preventive-based professionals such as EHOs, who are already deployed across the state, heavily involved in the management of the pandemic.

Throughout their tertiary training, EHOs receive risk communication knowledge and strategies. As this research has indicated, the risk communication to both professionals and the community was untimely and, in some cases, poor. Integrating EHOs into any future pandemic management would enhance public health outcomes and increase the understanding of the importance of such interventions by laypeople and policymakers. However, as this research has indicated, several challenges to this integration exist, and many of them lie in the training, promotion and retention of the EH professionals. These challenges include the low number of EHOs currently in the workforce, the depleting opportunities for tertiary studies in environmental health, the undervaluing of the profession, and competing priorities within the field. To address these challenges, the important public health role of the EHO needs to be made prominent. The Western Australian Local Government Association could partner with the EHA to promote the important role and benefit of environmental health and wider regulatory services to local political leaders. The peak professional body (EHA) needs to explore opportunities to provide advanced and high-level skills training to EH professionals, including advocacy, leadership and influencing skills to enable self-promotion. Additional undergraduate opportunities specific to environmental health need to be offered by the tertiary sector. LGAs need to consider more robust budgets to attract and retain EHOs, to improve staff morale, and to provide recognition and opportunities to encourage existing experienced staff to stay in the public sector.

The EHO profession was almost invisible during the COVID-19 pandemic response in WA, yet, despite some workforce issues, they have the necessary skills to assist under the authority afforded to them by the *Public Health Act 2016*. It is recommended that the WA Government invite representation from EHA to be members on public health emergency and public health cross-government regulatory working groups.

## 5. Conclusions

This research has demonstrated that the COVID-19 pandemic in WA had clear gaps in its processes for managing response and resilience to pandemics. A notable gap was the exclusion of a key public health professional, the EHO, who has appropriate powers under the legislation to assist, and who has a clear role in prevention and emergency management. EHOs also are familiar with working as regulatory, authorized officers. A better understanding of on behalf of government of the role of EHOs as a crucial component of overall public health response, and the integration of local government EHOs into the management of future public health challenges would improve the preparedness of the government of Western Australia to manage future public health emergencies.

This research took advantage of the opportunity to test the effectiveness of a relatively new body of legislation under real-world conditions in the form of COVID-19. This epidemic provided an opportunity to utilize a “natural experiment” to evaluate the performance of the state-wide environmental health response during public health emergencies that could never be replicated under simulation. The research provided insight into the professional challenges of EHOs associated with managing a pandemic and highlighted gaps in the approach of that the WA government used to manage the COVID-19 epidemic. Specifically, this research identified that EHOs who collectively would have added value to the pandemic management through regulatory performance, infection control processes, risk communication and access to communities across WA were overlooked, undervalued, and suffered burnout.

Policy recommendations to guide future public health emergencies through operational lines of authority, delegations and line management functions were offered to guide improvements within the WA health system. By implementing these suggested policy changes, a more efficient and integrated delivery of environmental health services during emergencies could be achieved. These changes would facilitate rapid access by the state government to a widely dispersed professional workforce that can respond effectively to implement infection control strategies, enforce quarantine measures, follow up contacts and communicate risks to the public and local business operators.

## Figures and Tables

**Table 1:** Demographic profile of survey participants.

Age	Female	Male	Unspecified	Total	Percentage
20–30	6	3	0	9	11.5%
31–40	13	9	1	23	29.5%
41–59	7	17	0	24	30.8%
>50	5	16	1	22	28.2%
Total	31	45	2	78	100.00%

**Table 2:** Additional roles during COVID-19 as nominated by EHOs.

Additional Role	Percentage Responded
Contract tracing	17.9%
Enforcement of quarantine/audits	15.4%
Education/risk communication	12.8%
Monitoring self-isolation	7.7%
Field hygiene/infection control	7.7%
Knowledge and experience	2.5%
Vaccination roll-out	2.5%
Decision making ability	2.5%
COVID testing	2.5%

**Table 3:** Macro themes and associated sub-themes.

Macro Theme	Sub Theme
Utilisation of workforce	Underappreciated and under-utilized Provision of advice Business as usual Specific COVID-19 related tasks Managing risks associated with “stay at home” orders
Potential to assist in future pandemics	Contact tracing Swabbing Appointed as leads
Challenges during COVID-19	Accessing timely and correct information Interpretation of advice Lack of community role models
Regulatory tools	Not used
Community needs	Consistent messaging
Workforce needs	Additional training/education Increasing profile Resourcing Legislation gaps

## Data Availability

Not applicable.
